# Myths and methodologies: Considerations for evaluating the time course of thermoregulatory adaptation during heat acclimation

**DOI:** 10.1113/EP091536

**Published:** 2024-06-13

**Authors:** Christopher J. Tyler, Sean R. Notley

**Affiliations:** ^1^ School of Life and Health Sciences University of Roehampton London UK; ^2^ Department of Defence Defence Science and Technology Group Melbourne Australia

**Keywords:** acclimatisation, constant‐stress, heat adaptation, isothermal, overload

## Abstract

Since the early 1900s, repeated heat exposure has been used as a method to induce physiological adaptations that enhance our ability to tolerate heat stress during athletic and occupational pursuits. Much of this work has been dedicated to quantifying the time course of adaptation and identifying the minimum duration of acclimation required to optimise performance or enhance safety. To achieve this, investigators have typically applied classical (constant load) heat acclimation, whereby 60–90 min exercise is performed at the same absolute or relative intensity in a hot environment for 3–24 days, with adaptations evaluated using an identical forcing function test before and after. This approach has provided a foundation from which to develop our understanding of changes in thermoregulatory function, but it has several, frequently overlooked shortcomings, which have resulted in misconceptions concerning the time course of adaptation. It is frequently suggested that most of the thermoregulatory adaptations during heat acclimation occur within a week, but this is an oversimplification and a predictable artefact of the experimental designs used. Consequently, the time course of complete human adaptation to heat remains poorly understood and appears to vary considerably due to numerous individual factors. The purpose of this communication is to highlight the key methodological considerations required when interpreting the existing literature documenting adaptation over time. We also propose potential means by which to improve the way we induce and quantify the magnitude of adaptation to expedite discovery.

## 1 INTRODUCTION

**FIGURE 1:**
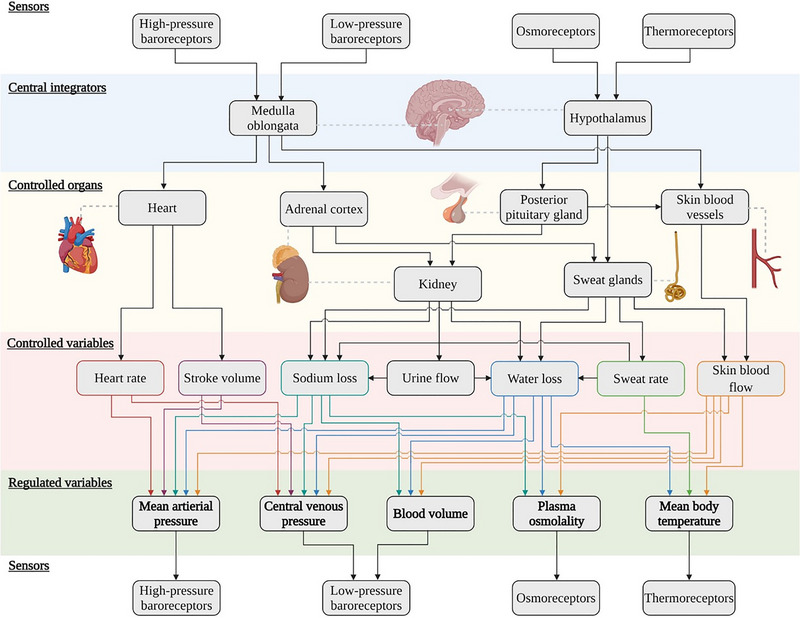
An overview of the major homoeostatic (regulatory) mechanisms activated during repeated heat exposure. Five regulated variables are illustrated, each with sensors (top and bottom rows), which provide feedback to the central integrators. These then modify the function of the controlled organs, which activate several physiological responses (the controlled variables). The figure is reproduced with permission from Taylor et al. (2020).

Humans have evolved the ability to acquire thermal tolerance, an ability that can attenuate the impairments in health, well‐being, performance and productivity often observed during heat stress. Thermal tolerance is acquired through physiological adaptations to several regulatory pathways (Figure 1), which occur in response to the repeated application of a thermal stimulus that sufficiently displaces body temperature (Taylor et al., 2020). That stimulus can arise naturally through climatic changes (acclimatisation) or artificially through planned heat exposures (acclimation).

Historically, classical (constant load) heat acclimation has typically involved prolonged (60–90 min) daily exercise performed at the same absolute or relative intensity in a hot environment (e.g., Horvath & Shelley, 1946; Robinson et al., 1943) with adaptations evaluated using an identical forcing function test prior to, and following, that treatment. This approach has provided a foundation from which to develop our understanding of heat adaptation, but it has several, frequently overlooked shortcomings which have, in our view, resulted in misconceptions concerning the time course of adaptation. The purpose of this communication is to describe these shortcomings, highlight gaps in our understanding, and propose ways to induce and quantify the magnitude of adaptation to improve our mechanistic understanding of the time course of heat acclimation. No attempt will be made to review general principles related to experimental design nor describe the physiological mechanisms surrounding heat adaptation, although we encourage reading of recent reviews on this topic (Taylor et al., 2020; Tyler et al., 2016).

## 2 HOW LONG DOES IT TAKE TO ADAPT DURING HEAT ACCLIMATION?

**FIGURE 2:**
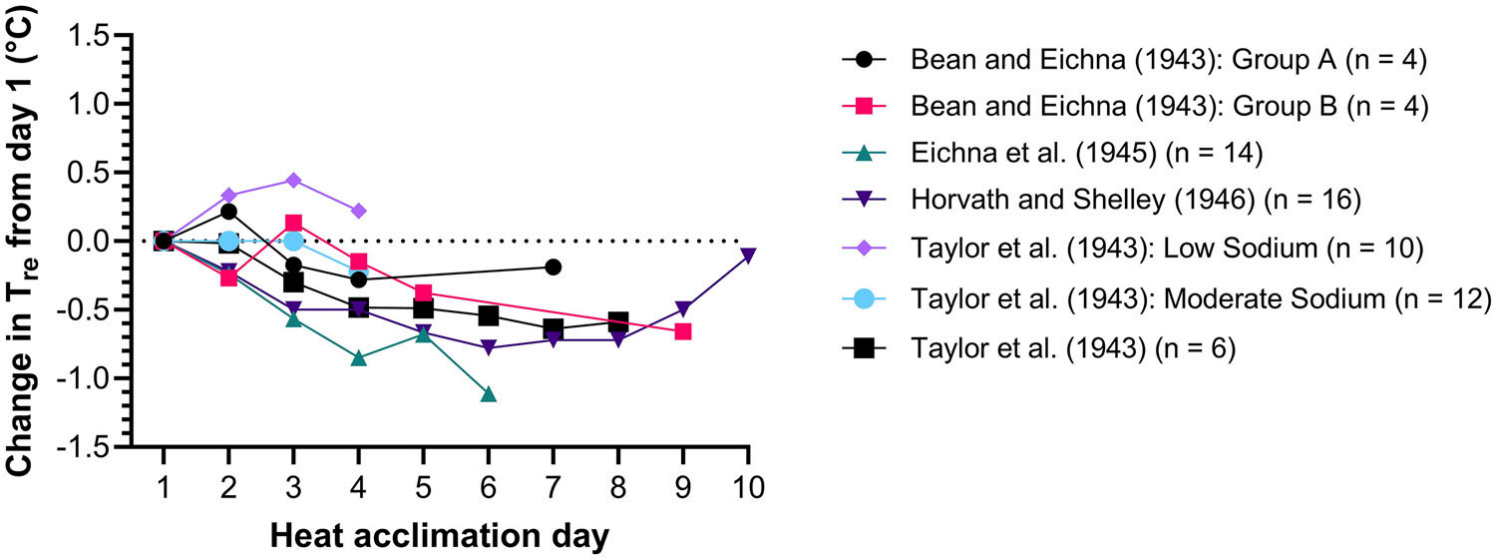
The change in end‐exercise rectal temperatures (*T*_re_) from the first day of constant‐stress heat acclimation reported in the seminal 1940s investigations (Bean & Eichna, 1943; Eichna et al., 1945; Horvath & Shelley, 1946; Taylor et al., 1943a, 1943b). Data from one study (Robinson et al., 1943), could not be plotted as the means for each session were comprised of different participants. All data points are group means, converted to metric units where required. Data were either obtained directly from the manuscript or by using figure‐reading software (PlotDigitizer.com).

Complete physiological adaptation to heat is a theoretical concept in which the physiological variables modified during acclimation all reach an adaptation plateau, and the time course of such adaptation has long interested researchers (Taylor et al., 1943). The time required to reach an adaptation plateau is likely to be variable‐specific (Tyler et al., 2016), but despite statements to the contrary, the time course of complete heat adaptation is unknown. Reductions in body temperature and heart rate are two ‘classic markers’ of heat adaptation (Sawka et al., 2011, p. 1897), although it has long been known that ‘one cannot define the process or detect differences in the degree of acclimatization between individuals by such simple measurements alone’ (Bean & Eichna, 1943, p. 146), and it has long been suggested that the majority of these reductions occur within about a week of acclimation (e.g., Pandolf, 1998; Sawka et al., 2011). The primary data thought to support this notion can usually be traced back to seminal studies from the 1940s (Bean & Eichna, 1943; Eichna et al., 1945; Horvath & Shelley, 1946; Robinson et al., 1943; Taylor et al., 1943a, 1943b), although they are rarely cited or scrutinised. For these seminal data to reflect the time course over which most of the possible heat adaptation occurs, a plateau would need to be observed and final‐day data would need to represent complete (100%) physiological adaptation. However, plateaux were rarely observed (Figure 2), and final‐day data are unlikely to represent complete adaptation due to the constant stress heat adaptation model applied in each investigation. The constant stress model fixes the work intensity, heat stress, or both to provide constant stress throughout the duration of acclimation, and although effective (Tyler et al., 2016), the overload decreases as adaptation progresses (Taylor, 2014). The decreasing overload results in an adaptation plateau below that of the physiological limit. It is therefore unlikely that most of the adaptation occurs within about a week of acclimation.

One way of providing a progressive overload is by using an isothermal (or controlled hyperthermia) heat acclimation protocol (Fox et al., 1963), which involves elevating and maintaining body core temperature at a pre‐determined temperature to provide an ever‐increasing impulse for adaptation (overload). If the protocol is sufficiently long in duration, adaptation should continue to the physiological limit (Taylor, 2014); however, this has not been confirmed. Further, investigators employing isothermal acclimation typically quantify the magnitude of adaptation by administering pre‐ and post‐constant‐stress heat stress tests (Tyler et al., 2016), which makes it impossible to evaluate the time course of adaptation for the ‘classic markers’ of adaptation. While some groups have reported data at baseline, during, and at the end of acclimation, these data are sparse and indicate that it can take longer than the 7 days previously stated to achieve greater than 75% of the total *observed* adaptation even when using an isothermal model (Magalhaes et al., 2010; Moss et al., 2020; Patterson et al., 2004). For example, 58% of the total observed adaption in resting rectal temperature was reported to occur on day 6 of an 11‐day protocol (Magalhaes et al., 2010), 97% on day 5 of a 10‐day protocol (Moss et al., 2020), and 62% on day 8 of a 22‐day regimen (Patterson et al., 2004). Once again, it is important to differentiate complete adaptation from the observed adaptation because despite providing an overloading stimulus, the magnitude of adaptation observed on the last day of the isothermal HA studies conducted to date is unlikely to represent complete adaptation. As such, there is a clear need for study designs involving longer‐term (>22 days) isothermal heat adaptation protocols and other protocols that may induce a progressive overload for adaptation (e.g., self‐selected intensity work or passive heating), ideally with daily evaluations of the magnitude of adaptation, to better characterise the time course of heat adaptation.

Mean data are often used to support conclusions regarding heat adaptation, but individual data highlight the extent of inter‐individual variability. For example, Robinson et al. (1943) concluded that the ‘rapid improvement in temperature regulation during the first 7 days amounted to about 80 percent of the entire improvement’ but the relative magnitude of adaption in end‐exercise rectal temperature observed on day 6 or 7 for each participant ranged from 44% to 73%. Despite differences in body mass (65–85 kg), participants exercised at the same intensity (3.5 miles^.^h^−1^ (~5.6 km^.^h^−1^) at 4%–5.6%) meaning that the resultant thermal impulse provided would have differed between individuals, and this may explain some of the variability. Isothermal approaches can provide a more comparable relative stress since the rise in body core temperature is clamped for all participants; however, as observed with adaptation to strength or endurance training programs, inter‐individual differences in the time course of adaptation responses are likely still extensive, due to factors such as acclimation status and history, intra‐ and inter‐individual variation in resting body core temperature (targeting a magnitude of change (e.g., +1.5°C) rather than a specific body core temperature may address this), and the rate of rise during the early stages of each session. Unfortunately, individual heat adaptation data from constant stress and isothermal heat acclimation are rarely reported and so the key variables that influence heat acclimation success, and their relative contributions, are currently unknown. Differences in variables such as age (Wagner et al., 1972), sex (Mee et al., 2015), hormonal profile (Wickham et al., 2021), ethnicity (Taylor, 2006), aerobic fitness (Pandolf et al., 1977), initial heat adaptation status (Taylor, 2006), previous acclimation history (Weller et al., 2007), the magnitude of the thermal impulse provided (Kissling et al., 2022) and the environment (Griefahn, 1997) may explain some of the variation and merit further study. Interrogating individual responses to any intervention, including heat acclimation, is associated with numerous statistical pitfalls so we encourage reading reviews on the topic before engaging in individual responder analysis (Atkinson et al., 2019).

## 3 CONSIDERATIONS FOR DESIGNING STUDIES TO BETTER UNDERSTAND THE TIME COURSE OF HEAT ADAPTATION

In the preceding discussion, we have described some potential limitations in our interpretation of existing data regarding the time course of adaptation. These limitations highlight the need for further research directed at characterising the time course of adaptation not only to improve understanding but also to enhance our ability to prepare for and tolerate heat exposure during occupational and athletic pursuits. The following subsections are aimed at proposing ways in which we can improve the way we induce and quantify the magnitude of adaptation to expedite discovery.

### 3.1 Protocol and session duration

While Patterson et al. (2004) reported that 22 days of heat acclimation was more effective than 8 days, it is unknown whether an adaption plateau had been reached and if so whether this represented complete or incomplete adaptation. This highlights the need for daily data from longer‐term heat adaptation protocols (>22 days). Further, most groups opt to conduct heat acclimation sessions lasting 60 or 90 min per day (Tyler et al., 2016), but it is plausible that to continue to induce adaptation during longer‐term acclimation, it may be necessary to extend this duration and increase the daily thermal impulse applied. For instance, assuming a starting body core temperature of 36.5°C, extending a 90 min isothermal exposure with a target body core temperature of 38.5°C (thermal impulse ∼150°C min) by 30 min would elicit an ∼40% increase in the daily thermal impulse applied (∼210°C min).

### 3.2 Thermal impulse overload

Isothermal heat acclimation studies usually raise and clamp body core temperature at ∼38.5°C, which ensures that the magnitude of body temperature displacement and thermal impulse increases as adaptation progresses (overload). Whether 38.5°C is sufficient to allow an individual to adapt to their physiological maximum remains uncertain, and a progressive overload in the absolute target temperature may be required. As a hypothetical example, body core temperature could be raised to, and clamped at, 38.0°C, 38.5°C and 39.0°C during weeks 1, 2 and 3 of acclimation, respectively. Provided those elevations are achievable, doing so will theoretically continue to elicit powerful compensatory physiological responses, as adaptation progresses; however, data from at least one experiment using this design suggest that there may not be an additive benefit of greater strain (Gibson et al., 2015). Further, if the overload is too high, this added strain may be counterproductive and potentially dangerous. The risk of heat‐related casualties during physically demanding work increases substantially during consecutive days of hot weather (Wyndham, 1965), and this may be, at least in part, due to reductions in thermoregulatory function, which exacerbate heat strain (Notley et al., 2018). The mechanism(s) explaining these effects remains unclear, although it seems probable that, much like endurance or strength training, there are limits to the overload that can be applied to optimise adaptation during heat acclimation, and this represents an important area of future study, particularly for athletes already engaged in demanding training.

### 3.3 Key variable(s)

Body core temperature is the most manipulated physiological variable during acclimation, but it is important to recognise that physiological adaptation originates from the displacement of our regulated variables (Figure 1). For thermoregulatory adaptation, that regulated variable is body temperature—the integrated temperature of central and peripheral tissues. For ease, this is often estimated as a mean body temperature calculated from the weighted sum of body core and mean skin temperature. Given that performing these calculations in real‐time is now possible, and skin temperature plays a role in the magnitude of adaptation (Regan et al., 1996), it would be advantageous to raise and clamp the mean body temperature, as opposed to just the body core temperature, during heat acclimation. As with body core temperature, the specific target mean body temperatures required to optimise adaptation are unknown.

### 3.4 Tests to quantify the magnitude of adaptation

In most instances, adaptation is quantified with heat stress tests involving exercise at fixed absolute work rates or relative percentages of peak aerobic power. For those interested in performance outcomes, this is often followed by a time trial or time‐to‐exhaustion test. While these approaches have merit when the emphasis is upon the extent to which heat adaptation might reduce physiological strain during occupational tasks or enhance athletic performance, they are less suited to understanding the thermoregulatory adaptations facilitating those benefits and for performing between‐group comparisons of the magnitude of adaptation. This is because these approaches will result in systematic differences in metabolic heat production and the subsequent requirement for heat loss (and drive for thermoeffector activation) among groups that differ in body size or aerobic fitness, which will occur independently from the heat acclimation treatment. Such between‐group differences may seem inconsequential if one's interest is to quantify between‐group differences in the change from pre‐ to post‐acclimation, but our ability to observe thermoregulatory adaptation appears to be partly dependent upon the combined exercise and environmental heat load applied (Poirier et al., 2015). As such, we would encourage investigators to instead consider applying tests involving increasing rates of metabolic heat production, which are standardised to body surface area (W/m^2^) to ensure the heat loss required to balance metabolic heat production is comparable between groups that differ in size or aerobic fitness (Notley et al., 2020). This involves modifying the external work rate to raise and clamp the rate of metabolic heat production, which is derived from the difference between external work rate and metabolic rate, with the latter being calculated in real‐time from measures of expired gas using a metabolic cart (Kenny & Jay, 2013).

Although standardising metabolic heat production to body size may be suitable for between‐group comparisons of thermoregulatory adaptation, it may be less than ideal for more mechanistic research (Taylor et al., 2020). For example, if one was interested in the way in which changes in thermoeffector function (sweating and cutaneous blood flow) facilitate increases in heat loss, exercise performed at a fixed external work rate, percentage of peak aerobic power, or metabolic heat production will result in a reduction in body temperature and the subsequent drive for effector activation following acclimation (Fox et al., 1963; Taylor, 2014). This makes it difficult to quantify the effects of adaptation from those associated with reduced physiological strain and may explain why findings related to the effects of heat acclimation on some variables (e.g., cutaneous vasomotor function) are equivocal within the literature (Tyler et al., 2016). To overcome that problem, one could standardise physiological strain across heat stress tests performed during acclimation. For example, to quantify the time course of sudomotor adaptation during heat acclimation, it would be wise to standardise the drive for sweating, which is best reflected by the mean body temperature (Taylor et al., 2020). To accurately show sudomotor adaptation in the presence of equivalent thermoafferent flow, it therefore becomes essential to administer heat stress tests eliciting the same change in mean (as opposed to body core) body temperature, calculating mean body temperature in real time using live body core and skin temperature data.

## 4 CONCLUSIONS AND RECOMMENDATIONS

We have attempted to highlight gaps in our understanding of the time course of thermoregulatory adaptation during heat acclimation and have proposed ways in which we can improve the way we induce and quantify the magnitude of adaptation. It is evident that the suggestion that most of the thermoregulatory adaptations during heat acclimation occur within a week is an oversimplification and a predictable artefact of the experimental designs used, and that the time course of human adaptation to heat remains unknown. To better understand the time course of heat adaptation, well‐designed, long‐term (>22 days) isothermal heat acclimation investigations are required. To expedite this discovery, we have also provided readers with an awareness of some methodological considerations that are often overlooked when designing experiments directed at quantifying the time course of heat adaptation. These considerations and our recommendations are summarised below:

- The magnitude of adaptation should not be reported as a percentage of the complete adaptation observed, as this reflects the proportion of adaptation to the stress provided rather than one's physiological maximum per se.
- Heat acclimation methods that provide a progressive overload for adaptation such as isothermal heat acclimation (controlled hyperthermia) are preferable over constant‐stress acclimation to avoid an adaption plateau; however, overloading the stimulus for adaptation can be counterproductive.
- Thermoregulatory adaptation is driven by displacement of the regulated variable, body temperature, which reflects the integrated temperature from both deep‐body and peripheral structures. It is therefore preferable to raise and clamp the mean body temperature, as opposed to the core temperature alone.
- Resting data are easier to collect than mean data; however, such data are more susceptible to non‐experimental stimuli and so thorough pre‐trial standardisation is required to ensure that differences represent adaptation rather than noise.
- Due to inter‐individual variability the key daily, data should be reported for each participant.
- Heat acclimation protocols can differ in duration, frequency and intensity—all of which will influence the thermal impulse provided. The optimal approach is unknown and will depend on the desired effect of the heat acclimation (e.g., complete vs. partial adaptation).
- Variables such as age, sex, ethnicity, body morphology, aerobic fitness, initial heat adaptation status, and previous acclimation or acclimatisation history may influence the time course of adaptation and so should be clearly reported and the topic of further study.
- When striving to perform meaningful comparisons of the magnitude of thermoregulatory adaptation among groups that differ in size or aerobic fitness, investigators should consider standardising the metabolic heat production per unit surface area or change in mean body temperature.

## AUTHOR CONTRIBUTIONS

Christopher J. Tyler conceived the manuscript. Christopher J. Tyler and Sean R. Notley wrote and reviewed the manuscript. Both authors have read and approved the final version of this manuscript and agree to be accountable for all aspects of the work in ensuring that questions related to the accuracy or integrity of any part of the work are appropriately investigated and resolved. All persons designated as authors qualify for authorship, and all those who qualify for authorship are listed.

## CONFLICT OF INTEREST

None declared.

## FUNDING INFORMATION

None.
